# A scoping review on advancements in noninvasive wearable technology for heart failure management

**DOI:** 10.1038/s41746-024-01268-5

**Published:** 2024-10-12

**Authors:** Niels T. B. Scholte, Annemiek. E. van Ravensberg, Abdul Shakoor, Eric Boersma, Eelko Ronner, Rudolf A. de Boer, Jasper J. Brugts, Nico Bruining, Robert M. A. van der Boon

**Affiliations:** 1 Erasmus Medical Center, Thorax Center, Department of Cardiology, Cardiovascular Institute, Rotterdam, the Netherlands; 2 Department of Cardiology, Reinier de Graaf Hospital, Delft, the Netherlands

**Keywords:** Heart failure, Hardware and infrastructure, Diagnosis

## Abstract

Wearables offer a promising solution for enhancing remote monitoring (RM) of heart failure (HF) patients by tracking key physiological parameters. Despite their potential, their clinical integration faces challenges due to the lack of rigorous evaluations. This review aims to summarize the current evidence and assess the readiness of wearables for clinical practice using the Medical Device Readiness Level (MDRL). A systematic search identified 99 studies from 3112 found articles, with only eight being randomized controlled trials. Accelerometery was the most used measurement technique. Consumer-grade wearables, repurposed for HF monitoring, dominated the studies with most of them in the feasibility testing stage (MDRL 6). Only two of the described wearables were specifically designed for HF RM, and received FDA approval. Consequently, the actual impact of wearables on HF management remains uncertain due to limited robust evidence, posing a significant barrier to their integration into HF care.

## Introduction

Heart failure (HF) impacts around 63 million individuals worldwide, significantly affecting patients and their caregivers^1^. Moreover, it places a significant strain on the healthcare system, primarily due to the necessity for frequent outpatient visits and recurrent hospitalizations^2^. This escalation in demand for services and resources is especially challenging in today’s healthcare environment, which is already facing issues with limited capacity, staff shortages and high workload^3^. Remote monitoring (RM) has been proposed as a solution to reduce this burden, with a recent meta-analysis highlighting its effectiveness showing that both invasive and non-invasive RM technologies can significantly lower mortality and hospitalization rates among HF patients^4^. However, the landscape of RM modalities is remarkably diverse, ranging from non-invasive blood pressure measurements to invasive hemodynamic sensors (e.g., CardioMems and Cordella device)^4–6^. Moreover, the adoption of RM technologies faces obstacles due to the absence of standardized methodologies and external validation^7^. These challenges contribute to a notable gap in determining the appropriate technology for specific patient categories. As a consequence, the present HF guidelines offer a limited endorsement for incorporating RM in the care of HF patients^8^.

Simultaneously to this development, there has been a rapid surge in a large array of (commercially available) health technology, including wearable devices ranging from smartwatches, rings and accessories incorporated into clothing^9^. Leveraging these wearable devices to monitor physiological variables offers a personalized and empowering experience for patients, that might become an important chain in modern HF-management. However, the efficacy of most of these wearables have poorly been studied^10^. Thus, as physicians increasingly embrace wearables for monitoring, critical questions persist regarding their safety, readiness, and validity^11^. Consequently, regulatory bodies have taken steps to ensure the safe and effective application of these devices for medical purposes. For instance, the European Union’s Medical Device Regulation (MDR) and U.S. Food and Drug Administration (FDA) have established classifications and guidelines to regulate wearable devices that may have medical applications^12,13^. Addressing these knowledge gaps and informing the HF community about the integration of these devices in clinical practice is of paramount importance. Therefore, we performed a comprehensive scoping review to provide an overview of all wearable devices currently being tested and used in HF management. This review aims to elucidate their functionalities, applications, and evaluate their developmental progress in the HF population by obtaining their MDR/FDA classification and by using the Medical Device Readiness Level (MDRL) framework^14^.

## Results

**Fig. 1:**
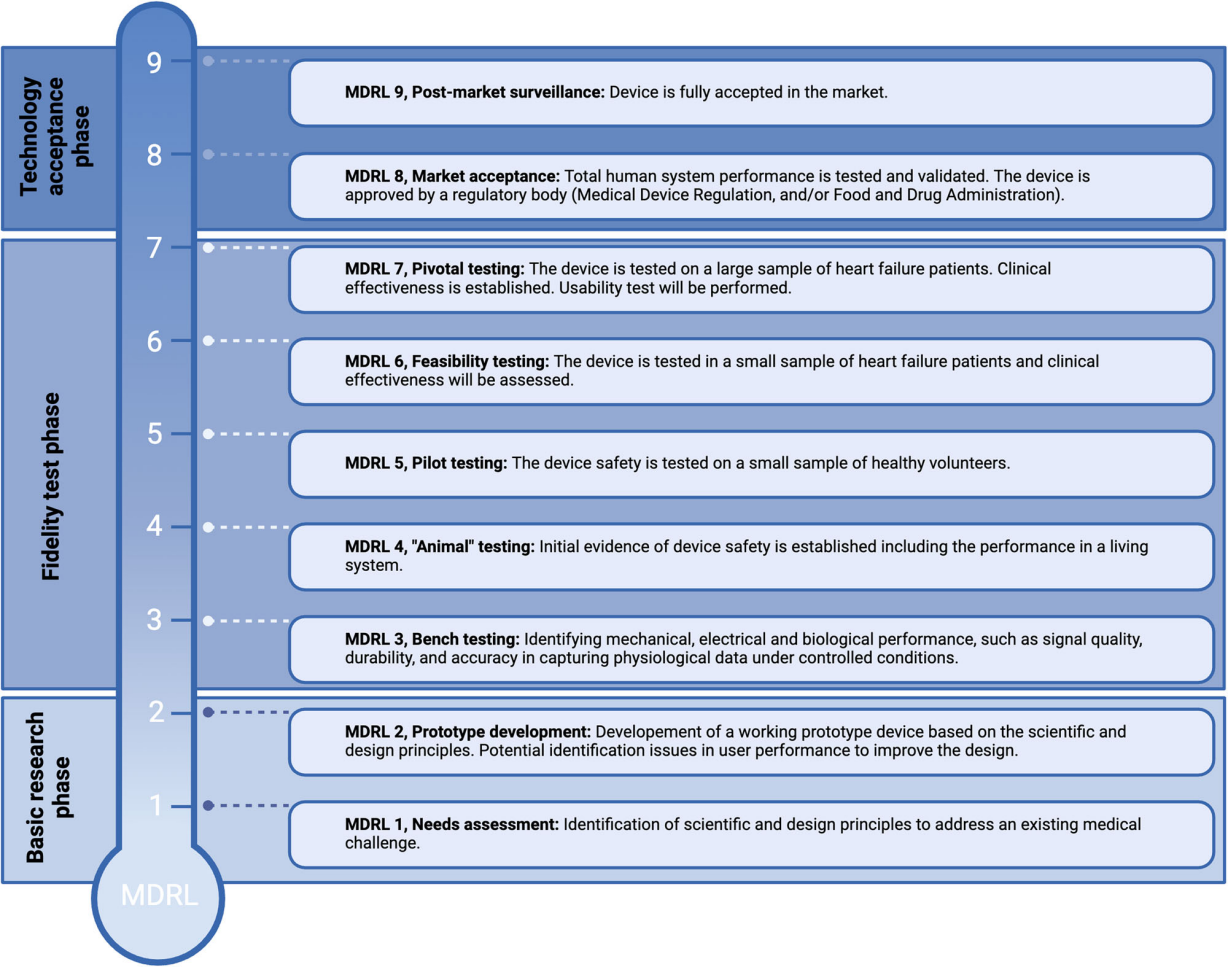
Description of the Medical Device Readiness Level (MDRL). In this figure, each stage of the Medical Device Readiness Level (MDRL) is briefly described. A more detailed description of each stage and MDRL itself can be found in the article of Ruiz Seva et al.^14^.

A total of 3112 articles were identified and after screening for eligibility, 99 studies were included, involving 13.879 patients (Fig. 1)^15–113^. A detailed overview of the characteristics of the included studies is provided in Table 1 and Supplementary Table 1. Over time, the number of studies including wearables has steadily increased. Most studies included wrist-worn devices (*n* = 43, 43.4%), such as accelerometers, followed by vests (*n* = 23, 23.2%) and hip-worn devices (*n* = 20, 20.2%). (Table 2) The majority of these studies were designed as prospective studies, accounting for 91.9% of the included studies, and were predominantly conducted in the United States, Western Europe, and Japan. Our search identified only 8 RCTs that involved wearable technology^26,28,36,38,41,45,78,107^. However, no articles were found that tested the effectiveness of a wearable device in a large randomized setting.

**Table 1:** Study characteristics

Authors, year	*n*	Country	Study type	Device	Type of device	Measure techniques	Functions	Medical grade	MDRL
Single measurement technique									
Alosco et al.^18^	96	USA	OBS	ActiGraph GT1M	Wrist-worn device	ACC	PA^a^	MDR C I, FDA C II	6
Alosco et al.^16^	65	USA	OBS	ActiGraph GT1M	Wrist-worn device	ACC	PA^a^	MDR C I, FDA C II	6
Alosco et al.^17^	57	USA	OBS	Actigraph GT1M	Wrist-worn device	ACC	PA^a^	MDR C I, FDA C II	6
Baril et al.^21^	50	CAN	OBS	Fitbit Flex	Wrist-worn device	ACC	PA	-	6
Blomqvist et al.^24^	605	SWE	OBS	ActiGraph GT9X	Wrist-worn device	ACC	PA^a^	MDR C I, FDA C II	6
Braun et al.^25^	61	DEU	OBS	wearable IMU (inertial measurement units) system (Rehagait®, Hasomed, Magdeburg, Germany	Ankles-worn	ACC	PA	-	6
Butler et al.^28^	69	USA	RCT	ActiGraph GT9X	Wrist-worn device	ACC	PA^a^	MDR C I, FDA C II	6
Da Silva et al.^30^	16	BRA	OBS	ActiGraph GT3X	Wrist-worn device	ACC	PA^a^	MDR C I, FDA C II	6
Dibben et al.^35^	22	GBR	OBS	GENEActiv, Activinsights Ltd.	Wrist- and waist-worn device	ACC	PA	-	5
Dibben et al.^36^	247	GBR	RCT	GENEActiv, Activinsights Ltd.	Wrist-worn device	ACC	PA	-	7
Dontje et al.^37^	68	NLD	OBS	SenseWear® Pro3 Armband	Wrist-worn device	ACC	PA	-	6
Edwards et al.^39^	190	USA	OBS	ActiGraph AM-7164	Wrist-worn device	ACC	PA^a^	MDR C I, FDA C II	6
Evangalista et al.^41^	38	USA	RCT	Sportline Pedometer Model 330	Hip-worn	ACC	PA	-	6
Floegel et al.^43^	27	USA	OBS	ActivPAL3c™ and Tractivity®	Wrist-worn device	ACC	PA	-	6
Floegel et al.^42^	29	USA	OBS	ActivPAL3c™ and Tractivity®	Wrist-worn device	ACC	PA	-	6
Fulcher et al.^44^	93	USA	OBS	ActiGraph GT1M	Wrist-worn device	ACC	PA^a^	MDR C I, FDA C II	6
German et al.^47^	58	USA	OBS	Kenz Lifecorder Ex	Hip-worn	ACC	PA	-	6
Güder et al.^48^	12	DEU	OBS	Polar M430	Wrist-worn device	ACC	PA	-	6
Holber et al.^52^	531	USA	OBS	SenseWear® Pro3	Wrist-worn device	ACC	PA	-	7
Howie-Esquivel et al.^53^	32	USA	OBS	Micro Care Timeliness Monitors	Hip- and ankle worn	ACC	PA	-	5
Izawa et al.^59^	261	JPN	OBS	Kenz Lifercorder	Hip-worn	ACC	PA	-	6
Izawa et al.^58^	477	JPN	OBS	Kenz Lifercorder	Hip-worn	ACC	PA	-	6
Jehn et al.^61^	55	DEU	OBS	StayHealthy Inc. RT3, AiperMotion 300 PfH, OMRON Walking Style Pro	Wrist-worn device	ACC	PA	-	5
Jehn et al.^60^	710	DEU	OBS^b^	AiperMotion 300 PfH	Hip-worn	ACC	PA	-	6
Klompstra et al.^66^	32	SWE	PT	DirectLife TracmorD, Philips	Wrist-worn device	ACC	PA	-	6
Klompstra et al.^65^	64	SWE	OBS^b^	ActiGraph GT9X	Hip-worn	ACC	PA^a^	MDR C I, FDA C II	6
Li et al.^69^	1140	USA	OBS	Actical, Philips	Patch	ACC	PA, Sleep^a^	FDA C II	6
Lin et al.^71^	124	GBR	OBS	Axivity AX3 (Axivity Ltd)	Vest	ACC	PA	-	6
McCarthy et al.^73^	20	USA	OBS	New Lifestyles NL-800	Hip-worn	ACC	PA	-	6
Melczer et al.^74^	17	HUN	OBS	ActiGraph GT3X	Wrist-worn device	ACC	PA^a^	MDR C I, FDA C II	6
Melin et al.^75^	60	SWE	OBS	ActiGraph GT3X	Wrist-worn device	ACC	PA^a^	MDR C I, FDA C II	6
Miyahara et al.^76^	70	JPN	OBS	Omron HJA-350IT	Hip-worn	ACC	PA	-	6
Nelson et al.^79^	93	USA	OBS	Actigraph GT3X	Wrist-worn device	ACC	PA^a^	MDR C I, FDA C II	6
O’Donnell et al.^80^	596	GBR	OBS	Axivity AX3	Wrist-worn device	ACC	PA	-	6
Okwose et al.^81^	20	GBR	OBS	Omron HJ-321-E	Hip-worn	ACC	PA	-	6
Omar et al.^82^	63	DNK	OBS	Actigraph GT3X	Wrist-worn device	ACC	PA^a^	MDR C I, FDA C II	6
Pozehl et al.^85^	182	USA	OBS	ActiGraph GT3X	Wrist-worn device	ACC	PA^a^	MDR C I, FDA C II	6
Prescher et al.^86^	155	DEU	OBS^b^	AiperMotion 300 PfH	Wrist-worn device	ACC	PA	-	6
Radhakrishnan et al.^87^	10	USA	OBS	Withings Go activity tracker	Wrist-worn device	ACC	PA	-	6
Rullman et al.^89^	94	SWE	OBS	Actigraph GT3X	Wrist-worn device	ACC	PA^a^	MDR C I, FDA C II	6
Schmidt et al.^92^	22	PRT	OBS	ActiGraph GT3X	Wrist-worn device	ACC	PA^a^	MDR C I, FDA C II	6
Schoemaker et al.^97^	22	USA	OBS	Stayhealthy Inc. RT3	Hip-worn	ACC	PA	-	5
Schwendinger et al.^93^	56	USA	OBS	GENEActiv, Activinsights Ltd.	Wrist-worn device	ACC	PA	-	6
Shen et al.^95^	40	CHN	OBS	ActiGraph GT3X	Wrist-worn device	ACC	PA^a^	MDR C I, FDA C II	6
Shiraishi et al.^96^	31	JPN	OBS	Active Style Pro HJA-750C	Waist-Worn	ACC	PA	-	6
Snipelisky et al.^99^	110	USA	OBS^b^	Kinetic Activity Monitors	Hip-worn	ACC	PA	-	6
van den Berg-Emons et al.^104^	5	NLD	OBS	Custom build system	Body- and hip-worn	ACC	PA	-	5
van den Berg-Emons et al.^105^	36	NLD	OBS	Custom build system	Body- and hip-worn	ACC	PA	-	6
Vetrovsky et al.^106^	26	CZE	OBS	Garmin vívofit	Wrist- and hip-worn device	ACC	PA	-	6
Vetrovsky et al.^107^	26	CZE	RCT	Garmin vívofit, Actigraph wGT3X-BT	Wrist-worn device	ACC	PA^a^	ActiGraph: FDA C II, MDR C I	Garmin: 6, ActiGraph: 6
Waring et al.^109^	50	USA	OBS	ActiGraph GT3X+	Wrist-worn device	ACC	PA^a^	MDR C I, FDA C II	6
Witham et al.^110^	82	GBR	OBS	Stayhealthy Inc. RT3	Hip-worn	ACC	PA	-	6
Young et al.^113^	100	USA	OBS	Actigraph wGT3X-BT	Hip-worn	ACC	PA^a^	MDR C I, FDA C II	6
Alvarez-Garcia et al.^19^	100	USA	RCT	ReDS™ Wearable System	Vest	B-IMP	CONG	FDA C II	6
Amir et al.^20^	50	ISR	OBS	ReDS™ Wearable System	Vest	B-IMP	CONG	FDA C II	6
Bensimhon et al. ^22^	108	USA	PT	ReDS™ Wearable System	Vest	B-IMP	CONG	FDA C II	6
Curtain et al.^29^	66	GBR	OBS	Sensinel CPM System by Analog Devices, Inc.	Patches	B-IMP	CONG	-	6
Lala et al.^68^	220	USA	OBS	ReDS™ Wearable System	Vest	B-IMP	CONG	FDA C II	6
Polcz et al.^84^	155	USA	OBS	ZOE®	vest	B-IMP	CONG	-	6
Ueno et al.^103^	12	JPN	OBS	ReDS™ Wearable System	Vest	B-IMP	CONG	FDA C II	6
Aamodt et al.^15^	10	NOR	OBS	CardioSet Edema Guard Monitor	Vest	B-IMP	CONG	-	6
Guo et al.^49^	66	CHN	OBS	BECG1200A, Thoth Medical Technology Co	Patch	ECG	HR	-	6
Kikuchi et al.^64^	10	JPN	OBS	hitoe®, TORAY	Vest	ECG	HR, Rhythm	-	5
Dagan et al.^31^	29	ISR	OBS	BB-613WP	Wrist-worn device	PPG	HR, SpO2, Respiratory Rate, PTT	-	6
Lin et al.^72^	40	TWN	POC	Prototype of Smart Clothes	Wrist-worn device	SCG	Cardiac Time Intervals	-	4
Combined measurement techniques									
Yates et al.^112^	29	USA	OBS	Actiheart, Camntech limited	Wrist-worn device	ACC, ECG	PA^a^	FDA C II	7
Smeets et al.^98^	36	BEL	OBS	Wearable Bioimpedance Monitor (IMEC)	Vest	ACC, ECG, B-IMP	CONG	-	5
Stehlik et al.^102^	100	USA	OBS	VitalPatch® RTM (VitalConnect®)	Patch	ACC, ECG, B-IMP, Thermometer	PA, HR^a^, HRV, RR interval, Skin humidity	FDA C II	Insufficient information
Kovisto et al.^67^	20	FIN	POC	Custom-build multi-sensor device	Multi-sensor device	ACC, ECG, SCG	HR, Rhythm, CAB	-	All: 3
Blockhaus et al.^23^	140	DEU	OBS	WCD ZOLL LifeVest®	Vest	ACC, ECG, SCG	PA	FDA C III	6
Burch et al.^26^	197	USA	RCT	WCD ZOLL LifeVest®	Vest	ACC, ECG, SCG	PA, HR^a^	FDA C III	PA:6, HR: 9
Burkhoff et al.^27^	1066	USA	OBS	WCD ZOLL LifeVest®	Vest	ACC, ECG, SCG	HR^a^, CAB	FDA C III	HR: 9, CAB: 5
Erath et al.^40^	671	USA	OBS	WCD ZOLL LifeVest®	Vest	ACC, ECG, SCG	HR^a^, Rhythm^a^, CAB	FDA C III	HR, Rhythm: 9; CAB: 5
Garcia et al.^45^	1013	FRA	OBS	WCD ZOLL LifeVest®	Vest	ACC, ECG, SCG	HR^a^	FDA C III	9
Hillmann et al.^51^	276	DEU	OBS	WCD ZOLL LifeVest®	Vest	ACC, ECG, SCG	PA, HR^a^	FDA C III	PA:6, HR: 9
Iliodromitis et al.^54^	77	DEU	OBS	WCD ZOLL LifeVest®	Vest	ACC, ECG, SCG	PA	FDA C III	6
Jungbauer et al.^62^	1353	DEU	OBS	WCD ZOLL LifeVest®	Vest	ACC, ECG, SCG	HR^a^	FDA C III	9
Röger et al.^88^	105	DEU	OBS	WCD ZOLL LifeVest®	Vest	ACC, ECG, SCG	HR^a^	FDA C III	9
Mlakar et al.^77^	24	SVN	OBS	prototype of multi- sensor wearable	Multi-sensor device	ACC, ECG, Thermometer	PA, HR, Rhythm, EE, Temperature, skin-humidity	-	Insufficient information
Solar et al.^101^	*NA*	ESP	POC	Wearable platform	Body- and hip-worn	ACC, ECG, Thermometer, Humidity sensor	PA, HR, HRV, Temperature, Skin humidity, Potassium, EE	-	4
Deka et al.^33^	30	USA	OBS	Fitbit Charge HR	Wrist-worn device	ACC, PPG	PA, HR^a^	MDR C IIa, FDA C II	PA: 6 HR: 7
Dorsch et al.^38^	83	USA	RCT	Fitbit Charge 2	Wrist-worn device	ACC, PPG	PA, HR^a^	MDR C IIa, FDA C II	All functions: 6
Gardner et al.^46^	39	USA	OBS	Nonin WristOx2 model 3150, Actisleep+	Wrist-worn device	ACC, PPG	PA^a^, Sleep, SpO2^a^	ActiGraph: FDA CII, MDR C I; Nonin: FDA C II	All functions: 5
Herkert et al.^50^	19	NLD	OBS	Fitbit Charge 2 & Mio Slice	Wrist-worn device	ACC, PPG	PA, HR^a^, EE	Fitbit: MDR C IIa, FDA C II	Fitbit: PA: 6, HR: 6, EE: 5; MioSlice: PA: 6, HR: 6, EE: 5
Nagatomi et al.^78^	30	JPN	RCT	Fitbit Inspire HR	Wrist-worn device	ACC, PPG	PA, HR^a^	MDR C IIa, FDA C II	PA: 6 HR: 6
Sohn et al.^100^	20	USA	OBS	Fitbit Charge 2	Wrist-worn device	ACC, PPG	PA	MDR C IIa, FDA C II	6
Vetrovsky et al.^108^	29	CZE	OBS	Withings Go, Fitbit Charge 2, Garmin vivofit, Garmin vivofit 3, Omron HJ-322U-E, smartLAB walk+	Wrist-worn device	ACC, PPG	PA	Fitbit: MDR C IIa, FDA C II	All devices: 6
Iqbal et al.^57^	*NA*	USA	POC	Custom-built wearable belt	Hip-worn	ACC, PPG, ECG, B-IMP	PA, HR, Sleep, Rhythm	-	All functions: 4
Iqbal et al.^56^	10	USA	POC	Custom-built wearable belt	Hip-worn	ACC, PPG, ECG, B-IMP	PA, HR, Sleep, Rhythm	-	All functions: 5
Wong et al.^111^	20	CHN	OBS	Biofourmis Everion	Armband	ACC, PPG, ECG, Thermometer, Barometer	PA, HR, HRV, Temperature, Blood pulse wave, SpO2, Respiratory Rate	-	Insufficient information
Darling et al.^32^	106	USA	OBS	Philips fluid accumulation vest	Vest	B-IMP, ECG	CONG	-	6
Pan et al.^83^	37	USA	OBS	Philips fluid accumulation vest	Vest	B-IMP, ECG	CONG	-	5
Sanchez-Perez et al.^90^	24	USA	POC	Multimodal sensing system	Multi-sensor device	B-IMP, microphone	CONG, Rhythm	-	All functions: 5
Li et al.^70^	76	CHN	OBS	WENXIN® device	Patch	ECG, Microphone	Rhythm, CAB	-	All functions: 5
Inan et al.^55^	45	USA	OBS	Prototype sensing patch	Patch	ECG, SCG	HF status (compensated vs. decompensated)	-	4
Shandhi et al.^94^	59	USA	OBS	Custom-built wearable patch	Patch	ECG, SCG	VO_2_	-	5
Kaneko et al.^63^	49	JPN	OBS	AUDICOR AM-RT	Patch	PPG, ECG	CONG, HR, CAB	-	All functions: 6
Di Rienzo et al.^34^	*NA*	ITA	POC	SeisMote	Vest	PPG, ECG, SCG	HR, HRV, PTT	-	All functions: 4
Savoldelli et al.^91^	15	ITA	POC	Hodwy Senior of Comftech©	Vest	PPG, ECG, Thermometer	HR, Rhythm, Temperature	-	All functions: 5

*NOR* Norway, *USA* United States of America, *ISR* Israel, *SWE* Sweden, *DEU* Germany, *AUT* Austria, *BRA* Brazil, *ITA* Italy, *GBR* United Kingdom, *NLD* The Netherlands, *FRA* France, *CHN* China, *JPN* Japan, *FIN* Finland, *TWN* Taiwan, *SVN* Slovenia, *DNK* Denmark, *PRT* Portugal, *CZE* Czech Republic, *OBS* Observational study, *RCT* Randomized controlled trial, *POC* Proof-of-concept, *PT* Pilot trial, *ACC* Accelerometry, *B-IMP* Bio-impedance, *ECG* Electrocardiography, *SCG* Seismocardiography, *PPG* Photoplethysmography, *PA* Physical activity, *HR* Heart rate, *HRV* Heart rate variability, *PTT* Pulse transit time, *EE* Energy expenditure, *CONG* Pulmonary Congestion, *(C)HF* (Chronic) heart failure, *MDR* Medical device regulations, *FDA* Food and drug administration.

^a^These functions are medical graded.

^b^These studies are sub-studies of randomized controlled trials in which a wearable is used as measuring device in both the intervention and the control arm.

**Table 2:** Summarized information per measurement technique

	Total	Accelerometer	Bio-impedance	ECG	PPG	SCG	Thermometer	Other techniques^c^
Single measurement techniques	*N* = 65	*N* = 53	*N* = 8	*N* = 2	*N* = 1	*N* = 1	*N* = 0	*N* = 0
Type of device, *n* (%)^a^								
Wrist-worn device	35 (53.8)	33 (62.3)	-	-	1 (100)	1 (100)	-	-
Hip-worn device	17 (26.2)	12 (22.6)	-	-	-	-	-	-
Vest	9 (13.8)	1 (1.8)	1 (12.5)	1 (50.0)	-	-	-	-
Patch(es)	3 (4.6)	1 (1.8)	7 (87.5)	1 (50.0)	-	-	-	-
Multi-sensor device	-	-	-	-	-	-	-	-
Other types^b^	6 (9.2)	5 (9.4)	-	-	-	-	-	-
Study type, *n* (%)								
Randomized controlled trials	5 (7.7)	4 (7.5)	1 (12.5)	-	-	-	-	-
Observational studies	57 (87.7)	48 (90.5)	6 (75.0)	2 (100)	1 (100)	-	-	-
Proof-of-concept	1 (1.5)	-	-	-	-	1 (100)	-	-
Pilot trial	2 (3.1)	1 (1.8)	1 (12.5)	-	-	-	-	-
Geographical region, *n* (%)								
Asia	9 (13.8)	5 (9.4)	1 (12.5)	2 (100)	-	1 (100)	-	-
Europe	26 (40.0)	24 (45.3)	2 (25.0)	-	-	-	-	-
North America	27(41.5)	23 (43.4)	4 (50.0)	-	-	-	-	-
Latin America	1 (1.5)	1 (1.8)	-	-	-	-	-	-
Middle Eastern	2 (3.1)	-	1 (12.5)	-	1 (100)	-	-	-

Complex measurement techniques	*N* = 34	*N* = 25	*N* = 7	*N* = 26	*N* = 13	*N* = 13	*N* = 5	*N* = 4
Type of device, *n* (%)^a^								
Wrist-worn device	8 (23.5)	8 (32.0)	-	1 (3.8)	7 (53.8)	-	-	-
Hip-worn device	3 (8.8)	3 (12.0)	2 (28.6)	3 (11.5)	2 (15.4)	-	1 (20.0)	1 (25.0)
Vest	14 (41.2)	10 (40.0)	3 (42.9)	14 (53.8)	2 (15.4)	10 (76.9)	1 (20.0)	-
Patch(es)	5 (14.8)	1 (4.0)	1 (14.3)	5 (19.2)	1 (7.7)	2 (15.4)	1 (20.0)	1 (25.0)
Multi-sensor device	3 (8.8)	2 (8.0)	1 (14.3)	2 (7.7)	-	1 (7.7)	1 (20.0)	1 (25.0)
Other types^b^	2 (5.9)	2 (8.0)	-	1 (3.8)	1 (7.7)	-	2 (40.0)	2 (50.0)
Study type, *n* (%)								
Randomized controlled trials	3 (8.8)	3 (12.0)	-	1 (3.8)	2 (15.4)	1 (7.7)	-	-
Observational studies	24 (70.6)	18 (72.0)	4 (57.1)	19 (73.1)	7 (53.8)	2 (15.4)	3 (60.0)	2 (50.0)
Proof-of-concept	7 (20.6)	4 (10.0)	3 (42.9)	6 (23.1)	4 (30.8)	10 (76.9)	2 (40.0)	2 (50.0)
Pilot trial	-	-	-	-	-	-	-	-
Geographical region, *n* (%)								
Asia	4 (11.8)	2 (8.0)	-	3 (11.5)	3 (23.1)	-	1 (20.0)	2 (50.0)
Europe	14 (41.2)	12 (48.0)	1 (14.3)	12 (46.2)	4 (30.8)	8 (61.5)	3 (60.0)	1 (25.0)
North America	16 (47.1)	11 (44.0)	6 (85.7)	11 (42.3)	6 (46.2)	5 (38.5)	1 (20.0)	1 (25.0)
Latin America	-	-	-	-	-	-	-	-
Middle Eastern	-	-	-	-	-	-	-	-

*PPG* Photoplethysmography, *ECG* Electrocardiography, *SCG* Seismocardiography.

^a^Type device: In some more than one wearable is used, and therefore this percentage does in some techniques not add up to 100%.

^b^Other types include: ankle-worn devices (*n* = 2), a bracelet worn on the upper arm (*n* = 1), body-worn devices (*n* = 3), and waist-worn devices (*n* = 3).

^c^Other techniques include: barometry (*n* = 1), humidity sensor (*n* = 1), microphone (*n* = 2).

### Single measurement techniques

**Fig. 2:**
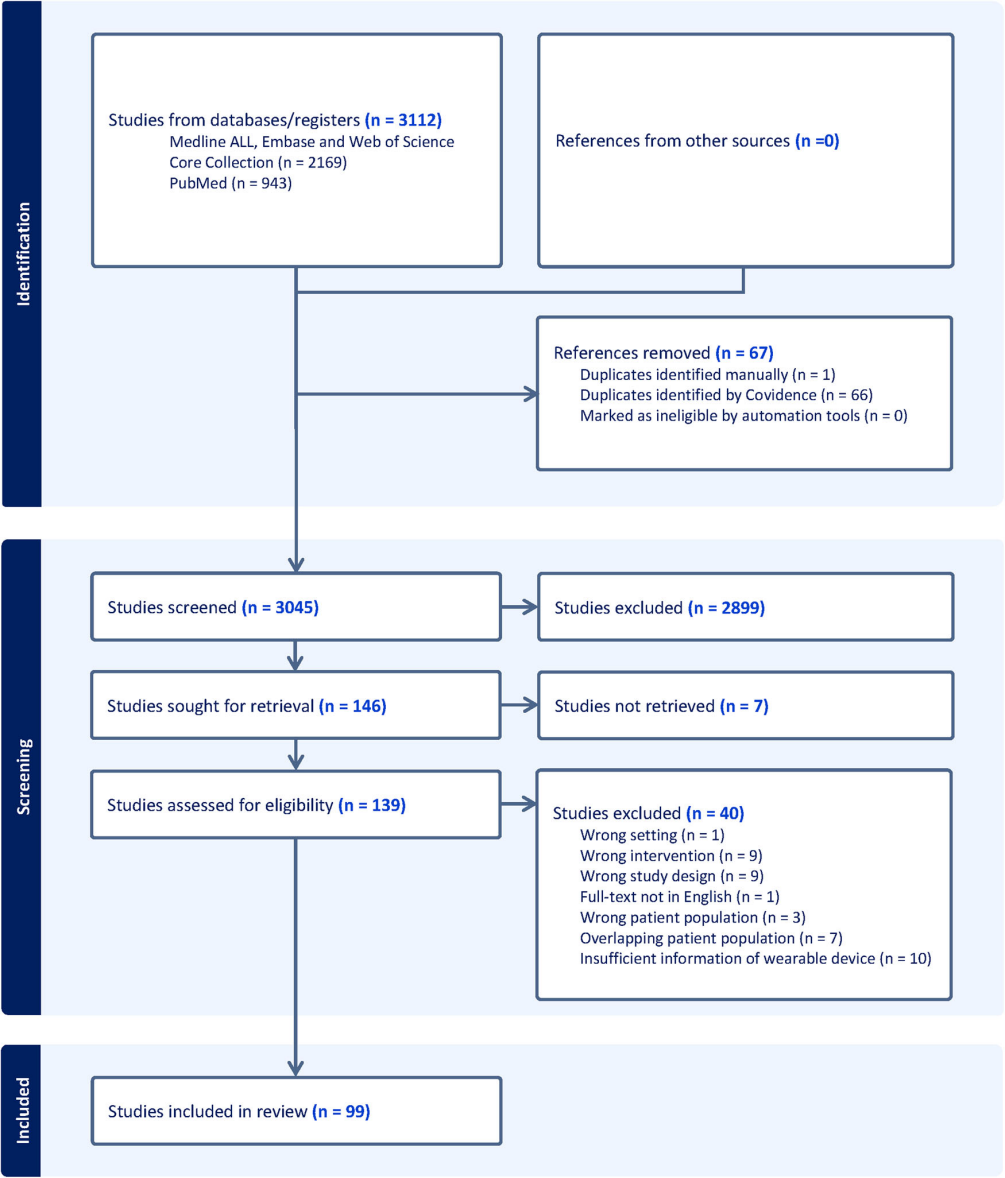
PRISMA flowchart. The flow diagram shows the number of studies identified and in- and excluded at the different stages of this scoping review.

In 65 studies included in this review, a wearable device using a single measurement technique was employed (Table 1). Among these, 53 used accelerometery for measuring PA, with the devices predominantly worn on the wrist (*n* = 33, 62.3%) or hip (*n* = 12, 22.6%). Notably, only the ActiGraph devices – the most frequent used accelerometery wearable (*n* = 53, 81.5%) - have a medical grade for measuring PA (MDR Class I and FDA Class II clearance). Most of these single measurement techniques wearables were considered having a MDRL of 6, indicating that most are in the feasibility phase for the intended purpose (Table 1 and Fig. 2). Most of these studies were observational in nature (90.1%) and only five were RCTs (7.7%). However, none of these studies primarily focused on the efficacy of RM through the use of accelerometers as they were either embedded in a home-based rehabilitation intervention or were part of a substudy in a larger clinical trial. A substantial number of included studies employing wearable accelerometers have demonstrated that HF patients exhibit lower PA levels compared to the healthy population, and experience worse outcomes when their PA is reduced^18,37,58,75,76,80,82,86,109^. Other studies have shown that PA, as measured by accelerometery, is associated with disease severity, as well as cognitive and executive function^16,17,25,44,71,99,104^. Additionally, three studies used accelerometers as part of a lifestyle intervention to motivate HF patients to increase their exercise^81,87,107^.

Apart from accelerometery, another technique that can be used in RM for HF is bio-impedance, which is briefly explained in Supplementary Table 2. This technique, which measures pulmonary congestion is used in eight studies (7.9%). The majority of these studies employed the ReDS ™ Wearable system, currently the only wearable with a medical grade (FDA Class II) for measuring pulmonary congestion^19,20,22,68,103^. Most studies using this technique were observational, except for one RCT and one pilot trial. Within the relatively small RCT (*n* = 100), a ReDS-guided treatment strategy was assessed in a hospital environment showcasing a reduction in clinical events at 30 days^19^. Currently, only one study of limited size (*n* = 10), described the use of this wearable in a remote environment, concluding that the use of such device is feasible in the first 30 days after discharge, and improves self-care behavior^15^. Consequently, the MDRL was estimated at 6 for all devices that rely solely on bio-impedance.

In two other studies, wearables that solely use ECG signals to measure heart rate and rhythm were utilized. In one study (*n* = 66), a patch (MDRL: 6) designed for extended wear was utilized as part of a Hospital-Community-Family-Based Telehealth Program^49^. In the other study (*n* = 11), the feasibility and safety of a telerehabilitation program were assessed in which a wireless ECG vest (MDRL: 5) was used to remotely monitor the HF patient during a cardiac rehabilitation session^64^. Lastly, one wearable, a prototype wearable with MDRL 4, used seismocardiography as technique to measure cardiac time intervals, which could contain valuable for HF monitoring, in a proof-of-concept study^72^.

### Complex measurement techniques

A total of 34 studies (34.3%) utilized wearable devices or a combination of wearables employing multiple measurement techniques. Most of the studies included were of observational nature (*n* = 24, 70.6%), with only three being RCTs (8.8%). Among these studies, the WCD Zoll LifeVest® (*n* = 9, 26.5%) and smartwatches from various brands (*n* = 9, 26.5%) were the most commonly used wearables. In the studies where the WCD Zoll LifeVest® was utilized, heart rate was the most often measured function^23,26,27,40,45,51,54,62,88^. The MDRL for the heart rate function was classified as 9, reflecting its core functionality in detecting life-threatening arrythmias and the ability to deliver therapy if necessary (medical grade: FDA Class III). This device can also be used to measure physical activity (MDRL: 6) and cardiac acoustic biomarkers (MDRL: 5)^23,26,27,40,51,54^. Multiple studies describe that the LifeVest can be used for RM of HF and thereby potentially improve clinical outcomes^23,27,62^ and provide valuable information regarding titration and treatment response of GDMT^51,54,62^.

Smartwatches and other wrist-worn devices form another group of wearables that utilize a combination of measurement techniques. All these devices incorporate accelerometers combined with either PPG, ECG or both. Among these wearables, Fitbit devices were the most frequently used (*n* = 6, 17.6%) and have a medical grade (MDR Class IIa, FDA Class II) for heart rate measurement. Within this group of devices, the MDRL ranged from 5 to 7, with the lowest MDRL for the functionality energy expenditure in both the Fitbit Charge 2 and Mio Slice devices, and the highest for heart rate in the Fitbit Charge HR. In studies employing these wrist-worn devices, most have demonstrated utility for clinical purposes in HF patients^108^, though these devices lacked sufficient accuracy in estimating the patients’ energy expenditure^50^. Additionally, other studies indicate that adherence to and usability of these devices are generally high^46,100^. There are a limited number of studies focusing on enhancing physical activity (PA) in HF patients^33,78^. One study focused on self-monitoring and self-management in recently admitted HF patients, which showed improved quality of life in the short-term; however, this effect did not persist to 12 weeks^38^.

Lastly, there are wearables that are specifically designed for the monitoring of HF patients. Those devices are described and used in 11 studies and ranged from prototype devices to more developed devices with MDRLs ranging from 3 to 6. These wearables utilize various combinations of measurement techniques as described in Table 1. The studies in which these less developed devices are described focus on the ability of measuring variables relevant to HF, such as pulmonary congestion and potassium blood content obtained from ECG signals^56,57,90,101^. Four studies showcased the ability of two wearables in tracking changes in pulmonary fluid status^32,83,90,98^. One of these studies demonstrated the usability of their device in a remote setting^32^. Two other studies demonstrate the correlation between cardiac acoustic biomarkers and absolute pulmonary artery pressures and HF state^63,67^.

## Discussion

In this scoping review, we evaluated the use of non-invasive wearables to monitor HF patients which resulted in the following main findings; (I) Currently, a diverse array of non-invasive wearables are used for monitoring HF patients, with a primary focus on physical activity assessment. Additionally, wearables encompassing ECG, PPG, SCG, and bio-impedance based sensors, facilitating the transmission of parameters such as heart rhythm, heart rate, and pulmonary congestion, have been studied. (II) Most of the wearables intended for RM of HF patients are currently in the feasibility phase (MDRL 6). However, many of these wearables were originally designed for different purposes or different populations and have been adapted for HF monitoring. (III) The majority of these devices are consumer-grade wearables and have not received FDA- or MDR approval for the monitoring of HF patients. (IV) While multiple devices demonstrated safety, reliability, and efficacy in small, predominantly observational, studies, there is a lack of large-scale RCTs to firmly establish the clinical advantages of wearable RM. Overall, our findings highlight the potential of wearables in RM for HF, but more so emphasize the need for further research to establish their clinical benefits before wider adoption and clinical implementation.

In the evolving landscape of remote HF management, the advent of non-invasive wearable technologies offers a promising solution to improve patient monitoring and care, while potentially easing the strain on healthcare systems^4,114^. In this study, accelerometers were identified as the wearable technology most thoroughly researched, mainly for tracking physical activity. Among them, the Actigraph accelerometer was the most frequently used, reaching a MDRL of 6 and achieving approval from both the FDA and MDR. However, the latter was not specifically for the RM of HF patients. The focus on accelerometers is particularly relevant given its availability and given the current reliance on the subjective New York Heart Association (NYHA) classification to assess functional status in HF patients, a method with a number of intrinsic limitations^115^. Our review has identified several studies describing a significant association between decreased physical activity, as measured by accelerometers, and key clinical outcomes, including reduced exercise capacity and quality of life, with some studies even linking it to increased mortality^58,76,79^. While accelerometers show promise in objectively measuring physical activity and potentially complementing traditional clinical assessments, the direct impact of such measurements on patient care, particularly in improving outcomes, remains an area requiring further investigation.

Current RM strategies surpass mere physical activity tracking, aiming primarily at identifying clinical deterioration, especially congestion, to avert hospital admissions or readmissions^116^. The challenge of detecting early congestion in HF patients, particularly during stages when symptoms are not present, is significant. This challenge is exacerbated by the limitations of physical examinations, laboratory markers, and patient-reported symptoms in predicting HF readmissions^117,118^. Such limitations underscore the urgent need for innovative, non-invasive methods capable of accurately assessing a patient’s volume status to guide diuretic treatment effectively. Our review has identified various wearable devices designed for the direct non-invasive monitoring of congestion. Notable innovations include the ReDS non-invasive vest and the CardioSet Edema Guard Monitor, both of which employ bio-impedance for direct congestion assessment. The ReDS system, in particular, has attracted the most interest with a considerable amount of evidence supporting its use. Currently, one RCT (NCT03586336) is in progress, exploring the ReDS system’s practicality and effectiveness in a real-world clinical context. Simultaneously, there is an emerging trend towards the development of more pragmatic wearables, such as smartwatches and small patches which are capable of estimating patients’ volume status or hemodynamic parameters associated with HF, including cardiac output and pulmonary wedge pressure^119^. The development of these indirect measurement techniques often involves the use of machine learning models integrating various variables, such as HR, HRV, pulse pressure timing (PPT), and physical activity (PA) in their estimation of the target parameter. Currently, these innovative approaches are still in the nascent stages of development, typically around MDRL 4–5, indicating a preliminary phase compared to the aforementioned devices^40,55^. Despite the potential of these ML-driven systems, the complexity and the black-box nature of the underlying algorithms pose challenges in clinical interpretation and acceptance^120^. To overcome these barriers and ensure the successful integration of these wearables into clinical practice, it is critical to advance clinical validation efforts. This includes conducting large-scale RCTs to ascertain their effectiveness and incorporating explainable AI approaches to demystify the decision-making processes of these technologies^121^.

Modern consumer wearables can perform measurements with a degree of accuracy comparable to regulated medical instruments. Consequently, the line between wearables designed for consumer use and those intended for medical applications is increasingly blurred. The general public is now more than ever using this data to monitor and improve their health^122^. A similar trend is evident in scientific research, as highlighted in this review, with the majority of the wearables under investigation being consumer-grade designed for different purposes or different populations and adapted for HF monitoring. While the use of consumer-grade wearables democratizes access to physiological data, it also poses challenges for healthcare providers. It’s essential to recognize that the majority of consumer-grade wearables (or part of their functionalities) have not undergone thorough validation, and even when they have, this validation has primarily been conducted on young and healthy individuals. Hence, clinicians should be cautious, recognizing that these wearables should not be employed for this purpose beyond research settings to safeguard patient well-being^123,124^. Moreover, the accuracy reported depends on the choice of the gold standard utilized. This underscores the importance of establishing standardized protocols and measures to conduct a robust assessment of the accuracy of these devices, as well as to define their operational limitations^10^. In this regard, it is striking that a MDRL below 6 hardly occurs in this category of wearables. In contrast, most devices specifically developed for HF patients were still in the prototype phase with the exception of the ReDS™ Wearable System which has a MDRL of 6^20,22,45,68,103^. When specifically developing a technology for HF patients, consideration was given from the start to which parameters could have clinical value, preliminary studies and validation were performed in the intended population. As such, the included prototypes a MDRL of 4 may therefore actually be further along in the process towards clinical application than a number of already commercially available wearables that have now been used on HF patients for the first time. This is also exemplified by the fact that the VitalPatch and ReDS™ Wearable System both have received FDA approval for the monitoring of HF patients, whereas none of the consumer-grade wearables have achieved such recognition^20,22,45,68,102,103^. If the results of both wearables are replicated in a larger RCT, the devices would obtain a MDRL of 7 or 8. However, the development, validation, and production of wearables tailored to specific purposes often entail higher costs, which can restrict their global adoption and availability. Although we did not include or report the costs associated with each wearable, our findings clearly illustrate this trade-off. Furthermore, they underscore the constraints of the MDRL, emphasizing that iterations should not only emphasize the significance of rigorous validation but also stress that it must be conducted within the target population.

A recent meta-analysis advocated for the use of RM for HF patients by showing that non-invasive RM of vital signs is associated with a significant reduction in the risk of first and total HF hospitalizations^4^. Integrating wearables into HF monitoring systems offers the possibility of significantly improving patient care through the continuous and objective tracking of physiological data. The enhanced connectivity of most wearable devices enables the real-time monitoring of changes in cardiac condition, providing a more immediate and comprehensive view of the patient’s health status. This continuous and up-to-date data acquisition has the potential to enhance the timeliness and predictive accuracy as compared to sporadic measurements^125^. The rationale behind this improvement lies in the ability to extract valuable insights from trends and effectively filter out daily fluctuations. However, this optimistic outlook on wearable technology integration comes with a caveat. Despite the ability to measure a vast array of physiological parameters, a critical question remains if these measurements tangibly benefit HF management and patient outcomes? The reality of healthcare innovation brings to light the complexity of translating data into actionable insights. While wearables hold the potential to foster proactive HF healthcare and encourage patient engagement and self-management, their effectiveness hinges on our ability to identify which measurements are clinically relevant and how they can be used to guide therapeutic decisions. Several challenges and opportunities remain to unlock the potential of RM with wearables. First, as described above, it is crucial to investigate the safety and effectiveness of wearables through randomized studies that not only include a larger patient population but also expand to encompass diverse geographical settings, including low-income countries, which were notably absent in our study^126^. Despite the proven safety and reliability of various devices in small observational studies, there is a notable lack of large RCTs confirming the clinical benefits of wearable RM. Current research often focuses on the devices’ ability to reliably transmit data, leaving their actual clinical impact largely unexplored due to the high cost of extensive trials. Execution of these trials are problematic because technological advancements in the field of wearables outpace the results of these trials. Novel trail designs are necessary to resolve this problem^127,128^. Furthermore, it is crucial to diversify study populations beyond the typical demographic of younger, white, wealthier individuals. Including varied socio-economic backgrounds and focusing on underrepresented groups will help ensure that wearable technologies can be equitably beneficial and tailored to the needs of all populations, particularly those in regions that are currently underserved by advanced medical technologies^129,130^. Rushing to integrate these devices into standard HF care without thorough testing in other demographic groups could result in flawed monitoring, as evidenced by numerous studies highlighting inaccuracies in PPG-derived SpO2 measurements among individuals with darker skin tones^131^. Furthermore, since these devices are not readily available to other populations, their integration may potentially exacerbate disparities in HF care^132^. To address this risk, it is critical to implement strategic measures such as expanding insurance reimbursement, shifting towards value-based payment models, and increasing public and private sector investments in wearable technology^125,133^. As we embrace wearable devices in our healthcare system, it is imperative to maintain an ongoing focus on equity concerns in order to address the pre-existing digital divide. Second, to facilitate reliable, long-term continuous measurements in a wearable form there is a need for further advancement in sensing and sampling technologies. Patients have well over 500.000 heartbeats and over 100.000 breaths a week. Noise, misinterpretation and false positives are thereby unavoidable. This will lead to extra care visits, which have to be weight against the benefits in resource restrained care systems. One way to accomplish this is by employing multimodal and/or multiplexed sensing, which involves using various transducer types which simultaneously measure different signals in a single wearable^134^. Third, further integration of wearables will create an overload of data that challenges the feasibility of the needed data infrastructure in hospitals. Not only does this require large-scale data storage, but the assessment of the resulting data will also be labor intensive when done by healthcare providers^125^. Consequently, the expanding use of wearables necessitates a corresponding increase in healthcare provider training programs. These programs should be designed not only to enhance data management skills but also to enable providers to make informed decisions based on the data collected by these devices. By contrast, the development of monitoring protocols, including alarm thresholds, will play an important role to mitigate this work. Additionally, the potential of cloud or fog computing, data mining, and machine learning in managing and interpreting the vast datasets generated by wearables has been highlighted^134^. These technologies could play a transformative role in automating data interpretation, thereby reducing the burden on healthcare providers and potentially enhancing the scalability of wearable technologies for RM of HF patients^135^. This could be further enhanced through the integration of feedback loops that would exclude the intervention of a healthcare provider (closed-loop). For example, a wearable device could allow for real time monitoring of the hemodynamic state of the patient and, in case of congestion, could offer an advice to increase the diuretic dosage or even give a subcutaneous bolus of the diuretic. Fourth, social acceptance of wearables must be ensured by informing users about the advantages and disadvantages and by integrating them into application ecosystems and health-care services. Last, the adoption of protocols for data safety and privacy with the establishment of an ethical regulatory framework for wearable data networks could further promote their use^136^.

Despite the various strengths inherent in this comprehensive scoping review, it is essential to acknowledge its limitations. Firstly, although the MDRL provides a comprehensive depiction of the developmental stage of a wearable device, it has a limitation when evaluating existing wearables, not specially designed for HF monitoring. Existing wearables do not progress to the entire MDRL framework and when tested in the target population automatically receive at least MDRL 5, as they are beyond the prototype phase. However, clinical value is not guaranteed merely by reaching this stage, as measurements might be unreliable in HF patients. Thus, a universally adapted scale is needed to prioritize the intended medical purpose for all stages of development, enabling a fair comparison of wearables’ readiness. Secondly, the inclusion of only English full-text articles and the omission of non-English articles and conference papers represent another limitation. Conferences and preprint servers play a crucial role in the technology and machine learning community. Consequently, this approach might have missed out on some novel developments and valuable insights from conference publications and non-English journals. However, conference papers may lack the same scrutiny of the peer-review process, potentially leading to biased results. Thirdly, it is important to note that both commercially available and medical-grade wearable activity monitors are constantly evolving, with new products being released each year. As a result, findings from studies conducted earlier in the data collection period may hold less relevance to researchers today. However, evolution of wearable technology does not invalidate the insights and knowledge gained from earlier studies. Instead, it adds to the growing body of research, allowing for comparisons, trend analysis, and identification of changes in technology and its impact on outcomes. Last, the objective of this scoping review was to describe the current evidence of the use of wearables in RM of HF patients. As such, we did not give a detailed description of the capabilities of each wearable, their underlying technique and separate functionalities.

In conclusion, this review evaluated the application of wearables in the RM of HF patients, highlighting a significant reliance on consumer-grade devices repurposed for medical studies. While a minority of wearables have been explicitly designed for HF monitoring and have even received FDA approval, the real value of these technologies in enhancing HF care remains under question. Especially, the paucity of large-scale RCTs underscores a critical gap in our understanding of the real-world benefits of wearables in managing HF. Addressing this question is essential for moving beyond “nice-to-have” gadgets to truly impactful tools in healthcare.

## Methods

We performed a scoping review with a systematic literature search of both randomized controlled trials (RCTs) and observational studies according to the Preferred Reporting Items for Systematic Reviews and Meta-Analyses for Scoping Reviews (PRISMA-ScR) guidelines^137^. The PRISMA-ScR checklist is provided in Supplementary Note 1. A prespecified local protocol was available.

### Search strategy and selection criteria

In collaboration with an expert librarian specialized in systematic searches a literature search was carried out on the 05/06/2024 including studies that were published up to that date, by using Embase, Medline Ovid, Web of Science and PubMed. Keywords used in the search included: *“heart failure”, “wearable electronic devices”, “telemedicine”*, and *“remote monitoring”*. The full search strategy is presented in Supplementary Note 2. Only peer-reviewed full original articles in the English language were included in our study. Studies were included if they contained any form of RM (or intended for RM) using a wearable sensor in chronic HF patients (NYHA class I-III or ambulatory class IV) aged 18 years or above. For this literature search, a wearable was defined as a connected electronic device that can be worn on the body as an accessory or embedded into clothing without burdening or hindering the wearer. In an effort to distinguish among the different types of wearables used in HF management, we define consumer-grade wearables as devices commercially available for general health monitoring, not specifically intended for medical use. Medical-grade wearables, including those used for research, are designed and validated for specific health monitoring purposes and are regulated by health authorities like the FDA or MDR. Non-invasive measure devices such as standard blood pressure monitors, electrocardiograms or handheld ultrasound were not defined as wearables. Additionally, studies about wearables (e.g., wearable cardioverter defibrillator) used for therapy only were excluded if there was no monitoring function used as well. Articles about the cost-benefit analysis of RM that include a wearable in their costs but do not provide detailed information about the used wearable were also excluded. The same applied for studies that solely described healthcare professionals’ and/or patients’ experiences with RM in general unless they provide specific, detailed information about the wearable device itself.

Three independent reviewers (A.R., A.S., and N.S.) independently performed screening of title and/or abstract to identify studies that potentially met the inclusion criteria. Hereafter, the full-text of each selected study was discussed in detail to decide upon the eligibility based on the inclusion and exclusion criteria. In case of any disagreement regarding eligibility and no consensus was reached the final decision was made by the last author (R.B.). If eligible studies described the same population, only the study with the longest follow-up or most recent publication containing the entire population was included, unless different outcomes of interest were studied in each article. Studies describing a subgroup of the same population were excluded. For title and/or abstract and full-text screening of the article, the online available systematic review tool “Covidence” was used.

### Data collection and extraction

The following information was extracted from the main study reports: author, year of publication, country, study name, study design, sample size, type of wearable, predictors the device can measure, sensor types of the device and endpoints of the study. Data was extracted in a predefined Microsoft Excel spreadsheet. Additionally, the medical certification for every device was collected from the online available databases of the Food and Drug Administration (FDA) and the Medical Device Regulation (MDR)^138,139^. In the United States, wearables used for medical purposes require FDA certification and wearables used in the European Union require MDR certification.

### Medical device readiness level

**Fig. 3:**
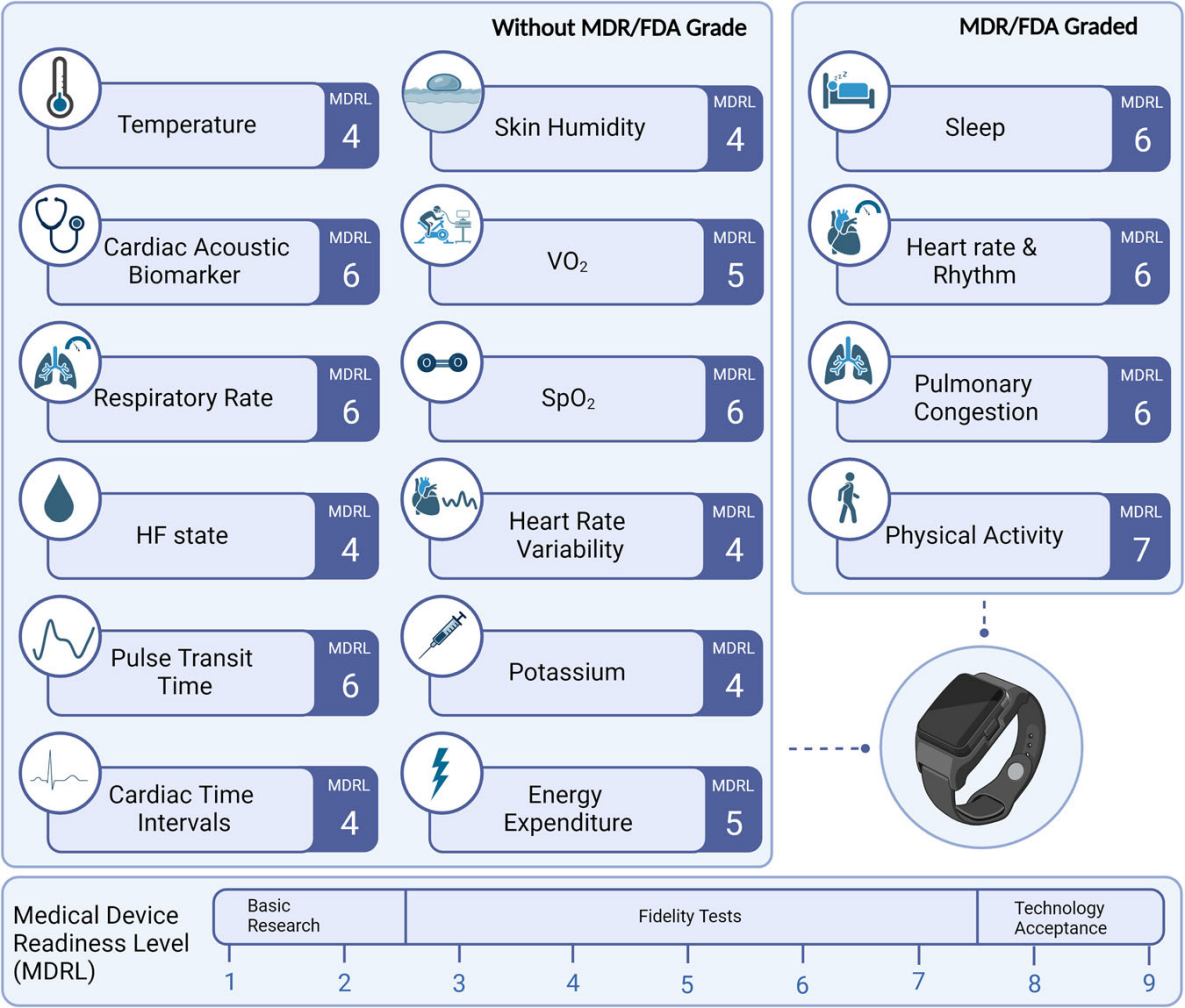
MDRL per variable. An overview of the variables utilized in various wearables in research for heart failure remote monitoring. For each variable, the Medical Device Readiness Level (MDRL) is provided. MDRL medical device readiness level, HF heart failure, SpO2 blood oxygen saturation, VO_2_ Oxygen uptake. MDRL presented in the figure represents the highest MDRL of that variable. Figure created using BioRender.

The Medical Device Readiness Level (MDRL), an adaption of the Technology Readiness Level, as proposed by Ruiz Seva et al.^14^ was used to assess the readiness of the wearables to be implemented in a clinical setting for HF monitoring. This measurement system has nine levels with general descriptions (Fig. 3). The MDRL levels ranged from 1, in which an existing medical challenge is addressed by identification of scientific and design principles, to MDRL 9 in which the wearable device is fully accepted in the market^14^. For this study, we specifically assessed the wearables’ readiness for HF monitoring. To ensure objectivity in determining the MDRL, each study was independently assessed by three researchers (N.S, A.S, R.B), and discrepancies were resolved through structured discussions to achieve consensus. The assessment was solely based on information extracted from the articles obtained through the systematic search. To capture variations in readiness based on specific monitoring functionalities (e.g., physical activity or heart rate monitoring), the MDRL was evaluated per function. This approach acknowledges that the readiness between the different functions may differ, especially when considering its application in the HF population.

## Supplementary information

Supplementary material

Figure Publication license

## Data Availability

The data underlying this article can be shared on reasonable request to the corresponding author.
