# Supplementary material for: A scoping review on advancements in noninvasive wearable technology for heart failure management
Source: NPJ Digit Med. 2024 Oct 12;7:279. doi: 10.1038/s41746-024-01268-5 (PMC11470936; doi:10.1038/s41746-024-01268-5)
Supplement: Supplementary file 1 — Supplementary material [file 41746_2024_1268_MOESM1_ESM.pdf]

Supplementary material

Content

Supplementary Note 1. PRISMA-ScR Checklist 2

Supplementary Note 2. Search strategy 4

Supplementary Table 1. Main conclusions of each included study 6

Supplementary Table 2. Explanations of different measurement techniques 11

Preferred Reporting Items for Systematic reviews and Meta-Analyses extension for Scoping Reviews (PRISMA-ScR) Checklist

SECTION	ITEM	PRISMA-ScR CHECKLIST ITEM	REPORTED ON PAGE #
**TITLE**			
Title	1	Identify the report as a scoping review.	#1
**ABSTRACT**			
Structured summary	2	Provide a structured summary that includes (as applicable): background, objectives, eligibility criteria, sources of evidence, charting methods, results, and conclusions that relate to the review questions and objectives.	#2
**INTRODUCTION**			
Rationale	3	Describe the rationale for the review in the context of what is already known. Explain why the review questions/objectives lend themselves to a scoping review approach.	#3
Objectives	4	Provide an explicit statement of the questions and objectives being addressed with reference to their key elements (e.g., population or participants, concepts, and context) or other relevant key elements used to conceptualize the review questions and/or objectives.	#3
**METHODS**			
Protocol and registration	5	Indicate whether a review protocol exists; state if and where it can be accessed (e.g., a Web address); and if available, provide registration information, including the registration number.	No registered protocol available
Eligibility criteria	6	Specify characteristics of the sources of evidence used as eligibility criteria (e.g., years considered, language, and publication status), and provide a rationale.	#4
Information sources*	7	Describe all information sources in the search (e.g., databases with dates of coverage and contact with authors to identify additional sources), as well as the date the most recent search was executed.	#4 anf fig. 2
Search	8	Present the full electronic search strategy for at least 1 database, including any limits used, such that it could be repeated.	Supplementary material
Selection of sources of evidence†	9	State the process for selecting sources of evidence (i.e., screening and eligibility) included in the scoping review.	#5
Data charting process‡	10	Describe the methods of charting data from the included sources of evidence (e.g., calibrated forms or forms that have been tested by the team before their use, and whether data charting was done independently or in duplicate) and any processes for obtaining and confirming data from investigators.	#5
Data items	11	List and define all variables for which data were sought and any assumptions and simplifications made.	#5
Critical appraisal of individual sources of evidence§	12	If done, provide a rationale for conducting a critical appraisal of included sources of evidence; describe the methods used and how this information was used in any data synthesis (if appropriate).	Not carried out
Synthesis of results	13	Describe the methods of handling and summarizing the data that were charted.	#5

SECTION	ITEM	PRISMA-ScR CHECKLIST ITEM	REPORTED ON PAGE #
**RESULTS**			
Selection of sources of evidence	14	Give numbers of sources of evidence screened, assessed for eligibility, and included in the review, with reasons for exclusions at each stage, ideally using a flow diagram.	#6, fig 2
Characteristics of sources of evidence	15	For each source of evidence, present characteristics for which data were charted and provide the citations.	#6, Table 1
Critical appraisal within sources of evidence	16	If done, present data on critical appraisal of included sources of evidence (see item 12).	Not carried out.
Results of individual sources of evidence	17	For each included source of evidence, present the relevant data that were charted that relate to the review questions and objectives.	Table 1, 2, Fig. 3
Synthesis of results	18	Summarize and/or present the charting results as they relate to the review questions and objectives.	#6 - #9
**DISCUSSION**			
Summary of evidence	19	Summarize the main results (including an overview of concepts, themes, and types of evidence available), link to the review questions and objectives, and consider the relevance to key groups.	#9 - 14
Limitations	20	Discuss the limitations of the scoping review process.	#15
Conclusions	21	Provide a general interpretation of the results with respect to the review questions and objectives, as well as potential implications and/or next steps.	#15, #16
**FUNDING**			
Funding	22	Describe sources of funding for the included sources of evidence, as well as sources of funding for the scoping review. Describe the role of the funders of the scoping review.	#16

JBI = Joanna Briggs Institute; PRISMA-ScR = Preferred Reporting Items for Systematic reviews and Meta-Analyses extension for Scoping Reviews.
* Where *sources of evidence* (see second footnote) are compiled from, such as bibliographic databases, social media platforms, and Web sites.
† A more inclusive/heterogeneous term used to account for the different types of evidence or data sources (e.g., quantitative and/or qualitative research, expert opinion, and policy documents) that may be eligible in a scoping review as opposed to only studies. This is not to be confused with *information sources* (see first footnote).
‡ The frameworks by Arksey and O'Malley (6) and Levac and colleagues (7) and the JBI guidance (4, 5) refer to the process of data extraction in a scoping review as data charting.
§ The process of systematically examining research evidence to assess its validity, results, and relevance before using it to inform a decision. This term is used for items 12 and 19 instead of "risk of bias" (which is more applicable to systematic reviews of interventions) to include and acknowledge the various sources of evidence that may be used in a scoping review (e.g., quantitative and/or qualitative research, expert opinion, and policy document).

From: Tricco AC, Lillie E, Zarin W, O'Brien KK, Colquhoun H, Levac D, et al. PRISMA Extension for Scoping Reviews (PRISMAScR): Checklist and Explanation. Ann Intern Med. 2018;169:467–473. doi: 10.7326/M18-0850.

Supplementary Note 2. Search Strategy

The search query for the systematic search was constructed to find all articles that meet the inclusion criteria. This resulted in the following search query:

Medline Ovid:

(exp * Heart Failure / OR ((heart OR cardiac* OR myocard*) ADJ3 (failure* OR Incometen* OR decompensat*)).ti. OR ((cardiac* OR cardiovasc*).ti. AND (exp Heart Failure/ OR ((heart OR cardiac* OR myocard*) ADJ3 (failure* OR Incometen* OR decompensat*)).ab,ti,kw.))) AND (Wearable Electronic Devices/ OR Fitness Trackers/ OR Telemetry/ OR Accelerometry/OR (wearable* OR smartwatch* OR smart-watch* OR ((activit* OR fitness* OR remote*) ADJ3 (monitor* OR track* OR sensor*)) OR telemet* OR acceleromet* OR pedomet* OR (body ADJ2 sensor*) OR (self* ADJ2 monitor*)).ab,ti,kw.) AND english.la.

Embase:

('heart failure'/exp/mj OR ((heart OR cardiac* OR myocard*) NEAR/3 (failure* OR Incometen* OR decompensat*)):ti OR ((cardiac* OR cardiovasc*):ti AND ('heart failure'/exp OR ((heart OR cardiac* OR myocard*) NEAR/3 (failure* OR Incometen* OR decompensat*)):ab,ti,kw))) AND ('wearable device'/de OR 'wearable sensor'/de OR 'wrist-worn device'/exp OR 'activity monitor'/de OR 'activity tracker'/exp OR telemetry/exp OR accelerometry/de OR accelerometer/exp OR pedometry/de OR (wearable* OR smartwatch* OR smart-watch* OR ((activit* OR fitness* OR remote*) NEAR/3 (monitor* OR track* OR sensor*)) OR telemet* OR acceleromet* OR pedomet* OR (body NEXT/2 sensor*) OR (self* NEXT/2 monitor*)):ab,ti,kw) NOT [conference abstract]/lim AND [english]/lim

Web of Science

(TI=((heart OR cardiac* OR myocard*) NEAR/2 (failure* OR Incometen* OR decompensat*)) OR (TI=(cardiac* OR cardiovasc*) AND TS=(((heart OR cardiac* OR myocard*) NEAR/2(failure* OR Incometen* OR decompensat*))))) AND TS=((wearable* OR smartwatch* OR smart-watch* OR ((activit* OR fitness* OR remote*) NEAR/2 (monitor* OR track* OR sensor*)) OR telemet* OR acceleromet* OR pedomet* OR (body NEAR/2 sensor*) OR (self* NEAR/2 monitor*))) AND DT=(article) AND LA=(English)

Pubmed:

(”Wearable Electronic Devices”[Mesh] OR ”Wearable Electronic Device*”[tiab] OR ”wearable*”[tiab] OR ”activity monitor*”[tiab] OR ”telemetry”[tiab] OR ”telemedicine”[tiab] OR ”remote monitoring”[tiab] OR “accelerometry”[tiab] OR “accelerometer*”[tiab] OR “fitness monitor*”[tiab] OR “activity tracker*”[tiab] OR “activity sensor*”[tiab] OR “pedometer*”[tiab] OR “smartwatch*”[tiab] OR “body sensor*”[tiab]) AND (”Heart Failure”[majr] OR ”Heart Failure”[Mesh])

Supplementary Table 1. Main conclusions of each included study

Authors, year	Study type	Measure techniques	Functions	Conclusion of study
Single measurement technique				
Alosco et al., 2012	OBS	Accelerometer	PA	Low PA is common in older adults with HF.
Alosco et al., 2014	OBS	Accelerometer	PA	Lower PA predicted worse cognition and cerebral perfusion 12-months later in HF.
Alosco et al., 2015	OBS	Accelerometer	PA	Reductions in daily PA predicted acute decline in attention/ executive function in HF.
Baril et al., 2019	OBS	Accelerometer	PA	The daily free-living step counts of patients with HF exhibiting NYHA class II versus class III symptoms are statistically different
Blomqvist et al., 2020	OBS	Accelerometer	PA	Single self-report question might be useful for high-specificity screening and identifying physically inactive patients
Braun et al., 2022	OBS	Accelerometer	PA	This study showed significant associations with some digital mobility outcomes and hand grip strength, suggesting that the (digital) assessment of mobility aspects could aid relevant information to presence and severity of HF in older adults
Butler et al., 2024	RCT	Accelerometer	PA	Accelerometer-based activity measures did not correlate with subjective or objective standard measures of health status and functional capacity in HF with preserved ejection fraction
Da Silva et al., 2013	OBS	Accelerometer	PA	PA assessed by accelerometer is associated with key cardiopulmonary exercise testing variables in HF.
Dibben et al., 2020	OBS	Accelerometer	PA	HF specific accelerometer intensity thresholds for inactivity and moderate-to-vigorous PA are lower than previously published thresholds based on healthy adults, due to lower resting metabolic rate and greater energy expenditure during daily living activities for HF patients
Dibben et al., 2023	RCT	Accelerometer	PA	Home-based cardiac rehabilitation didn't boost overall weekly PA, but changed behavior with increased weekday PA.
Dontje et al., 2014	OBS	Accelerometer	PA	The variance in daily PA in HF patients is considerable. Approximately half of the patients had a sedentary lifestyle.
Edwards et al., 2016	OBS	Accelerometer	PA	Sedentary behavior may not have detrimental HRQoL effects among congestive HF patients.
Evangalista et al., 2005	RCT	Accelerometer	PA	Pedometers are inexpensive and readily available to both clinicians and researchers. The results of this study suggest that they may be a valid indicator of exercise adherence in HF patients who participate in a home-based walking program.
Floegel et al., 2018	OBS	Accelerometer	PA	Hospitalized older HF patients were sedentary, which may contribute to decreased functional performance. PA after discharge may minimize this effect.
Floegel et al., 2019	OBS	Accelerometer	PA	Presentation of the feasibility and acceptability of continuous monitoring during hospitalization and at home.
Fulcher et al. , 2014	OBS	Accelerometer	PA	Findings show that PA is an independent predictor of cognitive function in HF.
German et al., 2021	OBS	Accelerometer	PA	PA variables were modestly correlated with measures of exercise capacity and not significantly with QOL.
Güder et al., 2022	OBS	Accelerometer	PA	Implementing a supervised HF-exercise program is feasible, safe, and may improve QOL and HF severity markers.
Holber et al., 2022	OBS	Accelerometer	PA	Although mood symptoms and LVEF were unrelated to objective PA, patients with higher step counts self-reported better HRQoL.
Howie-Esquivel et al., 2013	OBS	Accelerometer	PA	Immobility was pervasive as HF patients spent almost all of their time sitting or lying in bed despite their baseline ambulatory status and improved NYHA class.
Izawa et al., 2014	OBS	Accelerometer	PA	Poor mental health status may reduce PA. Attaining PA target values may improve mental health of CHF outpatients.
Izawa et al., 2013	OBS	Accelerometer	PA	Objectively measured step-count may be a prognostic indicator of mortality in Japanese outpatients with HF.
Jehn et al. , 2009	OBS	Accelerometer	PA	Accelerometers are reliable in measuring physical performance during the 6MWT in CHF patients.

Authors, year	Study type	Measure techniques	Functions	Conclusion of study
Jehn et al., 2013	OBS*	Accelerometer	PA	Tele-accelerometry is feasible in patients with CHF and output parameters are indicative of exercise capacity.
Klompstra et al., 2014	PT	Accelerometer	PA	Exergaming has the potential to increase exercise capacity in elderly, chronically ill cardiac patients.
Klompstra et al., 2022	OBS*	Accelerometer	PA	Exergaming interventions should target chronic HF patients with low social motivation and low level of light PA.
Li et al., 2021	OBS	Accelerometer	PA	Longer and more frequent objective napping predicted elevated future risk of developing incident HF.
Lin et al., 2022	OBS	Accelerometer	PA	Identification of patient characteristics associated with PA, which may identify those in need of interventions.
McCarthy et al., 2017	OBS	Accelerometer	PA	Brief exercise counseling may be an appropriate option to improve out-comes for stable patients with HF and may be tailored to fit different settings.
Melczer et al., 2016	OBS	Accelerometer	PA	With our method, based on the values received from the physical activity sensor implanted into the resynchronisation devices, changes in patients' health status could be monitored telemetrically with the assistance from the implanted electronic device.
Melin et al., 2016	OBS	Accelerometer	PA	The skewness in the intensity level of periods of high PA, was identified to be predictive of all-cause mortality independent of the Heart Failure Survival Score and peak VO2.
Miyahara et al., 2018	OBS	Accelerometer	PA	Low volume of PA postdischarge, predicts 6m postdischarge HF re-hospitalization in older HF patients.
Nelson et al., 2023	OBS	Accelerometer	PA	Older patients recently hospitalized for HF have low levels of PA and high levels of sedentary time, both of which may be targets for interventions. PA level was not significantly associated with objectively measured physical function, QOL, or cognition.
O'Donnell et al., 2020	OBS	Accelerometer	PA	Perceived and objectively recorded PA levels of CHF patients are lower than those of individuals without HF.
Okwose et al., 2019	OBS	Accelerometer	PA	The Active-at-Home-HF intervention is feasible, acceptable and effective for increasing physical activity in CHF. It may lead to improvements in quality of life, exercise tolerance and haemodynamic function
Omar et al., 2021	OBS	Accelerometer	PA	Among stable ambulatory patients with HFrEF, lower daily activity is associated with poorer cardiac index reserve and reduced cardiac index during exercise.
Pozehl et al., 2018	OBS	Accelerometer	PA	Highlighting the influence of race, anxiety, and attitudes toward exercise which have not been reported in previous exercise studies in patients with HF.
Prescher et al., 2016	OBS*	Accelerometer	PA	Tele-6MWT has a high predictive value with respect to HF hospitalization or all-cause mortality. Results were comparable with the prognostic impact of conventional 6MWT.
Radhakrishnan et al., 2020	OBS	Accelerometer	PA	To the best of the authors' knowledge, this usability and feasibility study is the first to report an Sensor-controlled digital game designed to improve HF self-management behaviors of older adults in their homes. Future research should consider several issues, such as user profiles, prior game-playing experiences, and network conditions most suitable for connected health interventions for older adults living in the community.
Rullman et al., 2020	OBS	Accelerometer	PA	These findings reinforce the importance of systemic circulatory factors linked to hemodynamic stress responses and inflammation in the pathogenesis and progress of HF disease.
Schmidt et al., 2020	OBS	Accelerometer	PA	Using accelerometer-derived data, HFpEF patients spent only a minority of their time involved in moderate-to-vigorous PA, which was the only PA pattern positively associated with prognostic indicators.
Schoemaker et al., 2013	OBS	Accelerometer	PA	There was good alignment of the MDD and MCID for the 6MWT, suggesting that clinically meaningful change is approximately 32 meters. However, the calculated MCID was substantially less than measurement error as represented by the MDD, indicating that the MCID was underestimated in this sample or that daily activity may be robust to change in overall disease status.

Authors, year	Study type	Measure techniques	Functions	Conclusion of study
Schwendinger et al., 2021	OBS	Accelerometer	PA	Due to substantial inter-individual variability in PA timing, accelerometers should be worn throughout waking time.
Shen et al., 2017	OBS	Accelerometer	PA	The wearable Holter-accelerometer data could help to identify impaired chronotropic response to PA in HF patients.
Shiraishi et al., 2021	OBS	Accelerometer	PA	An accelerometer could complement the KCCQ results in accurately assessing the physical activity in HF patients immediately after hospitalization, albeit itscorrelation with CPX was at most moderate
van den Berg-Emons et al., 2001	OBS	Accelerometer	PA	The results show how activities associated with mobility during everyday life may be restricted in people with CHF.
van den Berg-Emons et al., 2005	OBS	Accelerometer	PA	The results indicate that knee torque is associated with the level of movement-related everyday activity in people with CHF and that quality of life is mediated by nonphysiological factors.
Vetrovsky et al., 2020	OBS	Accelerometer	PA	Quarantine due to COVID-19 had a detrimental effect on the level of habitual PA in HF patients.
Vetrovsky et al., 2024	RCT	Accelerometer	PA	Whereas the lifestyle intervention in patients with HF with reduced ejection fraction improved daily steps by about 25%, it failed to demonstrate a corresponding improvement in functional capacity. Further research is needed to understand the lack of association between increased physical activity and functional outcomes.
Waring et al., 2017	OBS	Accelerometer	PA	Physical inactivity is related to 30-day all-cause readmissions for HF.
Witham et al., 2006	OBS	Accelerometer	PA	Six-minute walk distance predicts a small amount of the variance in daily activity, but the majority of variance in daily activity remains unexplained and requires further investigation.
Young et al., 2017	OBS	Accelerometer	PA	The present findings suggest that an accelerometer is a feasible and reliable measure of habitual PA in rural HF patients over time.
Alvarez-Garcia et al., 2024	RCT	Bio-impedance	Pulmonary congestion	A ReDS-guided strategy to treat congestion improved 1-month prognosis postdischarge in this proof-of-concept study, mainly because of a decrease of the number of HF readmissions.
Amir et al., 2017	OBS	Bio-impedance	Pulmonary congestion	ReDS-guided management has the potential to reduce HF readmissions in patients recently discharged.
Bensimhon et al.	PT	Bio-impedance	Pulmonary congestion	ReDS-guided management was associated with significant decongestion but not a reduction in HF readmissions in this sample.
Curtain et al., 2024	OBS	Bio-impedance	Pulmonary congestion	A non-invasive device was able to detect changes in congestion in patients with HF receiving decongestion therapy and patients having fluid removed at haemodialysis. The cardiopulmonary management device, which measures multiple parameters, is a potentially useful tool to monitor patients with HF to prevent hospitalizations.
Lala et al., 2021	OBS	Bio-impedance	Pulmonary congestion	ReDS-guided HF therapy during rapid follow-up after HF hospitalization may be associated with lower risk of 30 day readmission
Polcz et al., 2022	OBS	Bio-impedance	Pulmonary congestion	There was no correlation between ZOE® and PCWP
Ueno et al. , 2022	OBS	Bio-impedance	Pulmonary congestion	the lung fluid level varied considerably in patients with chronic HF following clinical stabilization
Aamodt et al., 2020	OBS	Bio-impedance	Pulmonary congestion	Daily lung impedance measurement and symptom diary feasible in HF monitoring.
Guo et al., 2019	OBS	ECG	HR	The Hospital-Community-Family-based telehealth program is feasible and provided researchers with evidence of remote hierarchical management for patients with CHF.
Kikuchi et al., 2021	OBS	ECG	HR, Rhythm	home-based cardiac rehabilitation using a telerehabilitation platform may be an option for patients unable to access center-based cardiac rehabilitation.
Dagan et al., 2022	OBS	PPG	HR, SpO2, Respiratory Rate, PTT	We show that physiological changes during diuresis in HF patients can be categorized into subgroups sharing similar response trends, making noninvasive monitoring a potential key to personalized treatment in HF.
Lin et al., 2018	POC	SCG	Cardiac Time Intervals	Features from the CHAMP system are correlated with myo-cardiac functions obtained from actual HF patients.

Authors, year	Study type	Measure techniques	Functions	Conclusion of study
Combined measurement technique				
Yates et al., 2017	OBS	Accelerometer, ECG	PA	Innovative rehabilitation nursing practice strategies are needed to assist patients in gaining the knowledge and skills to be more active and adhere to PA recommendations.
Smeets et al., 2020	OBS	Accelerometer, ECG, Bio-impedance	PA	The wearable bioimpedance device was able to track changes in fluid status during hospitalization and is a convenient method to assess the efficacy of decongestion therapy during hospitalization.
Stehlik et al., 2020	OBS	Accelerometer, ECG, Bio-impedance, Thermometer	PA, HR, HRV, RR interval, Skin humidity	Multivariate physiological telemetry from a wearable sensor can provide accurate early detection of impending rehospitalization.
Blockhaus et al., 2022	OBS	Accelerometer, ECG, SCG	PA	WCDs may be used as a telemonitoring and intervention tool in patients with reduced LVEF. Specific patient groups may benefit from guidance from their treating physician regarding physical activity.
Burch et al., 2020	RCT	Accelerometer, ECG, SCG	PA, HR	Similar in-clinic 6MWT results for clinician-guided and WCD-guided patients across objective distances.
Burkhoff et al., 2020	OBS	Accelerometer, ECG, SCG	HR, CAB	Cardiac acoustic biomarkers assess parameters associated with clinical status, and cardiovascular physiology in HF.
Erath et al., 2020	OBS	Accelerometer, ECG, SCG	HR, Rhythm, CAB	This prospective international registry showed that an algorithm incorporating CABs and HR data detected HF events 30 days in advance of the event in patients with HFrEF during first 3 months after hospital discharge.
Garcia et al., 2023	OBS	Accelerometer, ECG, SCG	HR	Dynamic monitoring of nocturnal HR may allow timely identification of impending cardiovascular events.
Hillmann et al., 2021	OBS	Accelerometer, ECG, SCG	PA, HR	Heart rate variability was an independent predictor for LVEF improvement and could serve as an early indicator of treatment response.
Iliodromitis et al., 2023	OBS	Accelerometer, ECG, SCG	PA	The WCD provides useful information regarding patient PA and may be additionally utilized for early HF treatment adjustment
Jungbauer et al., 2019	OBS	Accelerometer, ECG, SCG	HR	Remote HR monitoring may help to adequately titrate GDMT, thus improving clinical outcomes in HF patients.
Kovisto et al., 2022	POC	Accelerometer, ECG, SCG	HR, Rhythm, CAB	Three features indicating for decompensated HF in a serial-measurement scenario: increase root-mean-square strength, increase in S3 signal strength, and decrease in signal stability around S1.
Röger et al., 2018	OBS	Accelerometer, ECG, SCG	HR	The WCD as an external monitoring system contributed important infor-mation to optimize device selection in patients that needed ICD implantation.
Mlakar et al., 2018	OBS	Accelerometer, ECG, Thermometer	PA, HR, Rhythm, EE, Temperature, skin-humidity	The paper strongly points to the possibility of using patient reported outcomes as primary end-points in future trials.
Solar et al., 2013	POC	Accelerometer, ECG, Thermometer, Humididty sensor	PA, HR, HRV, Temperature, Skin humidity, Pottasium, EE	The platform monitors all the required parameters from sensors, collects and processes the data in a mobile platform and sends the data to a server. Specifically, the present solution monitors the electrocardiogram (ECG), potassium blood content (obtained from ECG), average energy expenditure evaluation through activity recognition, skin temperature and sweating.
Deka et al., 2018	OBS	Accelerometer, PPG	PA, HR	Wrist-worn devices can be useful for monitoring exercise adherence and PA in HF patients in a community setting.
Dorsch et al., 2021	RCT	Accelerometer, PPG	PA, HR	The adaptive mobile app intervention, improved the MLHFQ at 6 weeks but did not sustain its effects at 12 weeks.
Herkert et al., 2019	OBS	Accelerometer, PPG	PA, HR, EE	Both activity trackers demonstrated low accuracy in estimating EE in cardiac patients and poor performance to detect within-patient changes..
Nagatomi et al., 2022	RCT	Accelerometer, PPG	PA, HR	A comprehensive home-based cardiac rehabilitation programme using ICT for HF patients with physical frailty improved exercise tolerance and lower extremity muscle strength.

Authors, year	Study type	Measure techniques	Functions	Conclusion of study
Sohn et al., 2020	OBS	Accelerometer, PPG	PA	The majority of subjects maintained a high adherence to wearing the activity tracker.
Vetrovsky et al., 2019	OBS	Accelerometer, PPG	PA	While none of the tested activity monitors meet specific validity thresholds, most perform well enough to serve as useful tools for clinicians to motivate CHF patients to increase their walking activity.
Gardner et al., 2016	OBS	Accelerometer, PPG,	PA, Sleep, SpO2	Patients were able to use all of the devices and they rated the usability of all the devices higher than expected. Our study provides support for at-home patient-collected physiologic and subjective state data.
Iqbal et al., 2022	POC	Accelerometer, PPG, ECG, Bio-impedance	PA, HR, Sleep, Rhythm	Using different sensors embedded in a wearable belt. This wearable is able to continuously measure and monitor in real time all mentioned parameters in different conditions.
Iqbal et al., 2024	POC	Accelerometer, PPG, ECG, Bio-impedance	PA, HR, Sleep, Rhythm	A telehealth monitoring system has been developed that allows the continuous and real-time monitoring of parameters that are significant for HF. These parameters include transthoracic impedance, ECG, heart rate, and activity status.
Wong et al., 2022	OBS	Accelerometer, PPG, ECG, Thermometer, Barometer	PA, HR, HRV, Temperature, Blood pulse wave, SpO2, Respiratory Rate	HF with reduced ejection fraction medication escalation with remote monitoring appeared feasible.
Sanchez-Perez et al., 2022	POC	Bio-impedance, microphone	Pulmonary congestion, Rhythm	The system is suitable for detecting changes in pulmonary fluid status and capturing high-quality respiratory signals and lung sounds.
Darling et al., 2017	OBS	ECG, Bio-impedance	Pulmonary congestion	Patients discharged after acute decompensated HF can measure and transmit daily transthoracic bioimpedance using a FAV-mobile phone dyad. Algorithms analyzing thoracic bioimpedance may help identify patients at risk for recurrent HF events after hospital discharge
Pan et al., 2023	OBS	ECG, Bio-impedance	Pulmonary congestion	Potential for predicting acute decompensated HF from single-channel ECG recordings obtained from outpatients, enabling timely warning signs of HF.
Li et al., 2020	OBS	ECG, Microphone	Rhythm, CAB	Heart sounds and ECG signal index EMAT contributes to the diagnosis of EF <50%, especially in patients with inconclusive NT-pro-BNP.
Inan et al., 2018	OBS	ECG, SCG	HF status (compensated vs. decompensated)	Wearables recording cardiac function and machine learning algorithms can assess HF states by analyzing cardiac response to sub-maximal exercise.
Shandhi et al., 2020	OBS	ECG, SCG	VO_2	Wearable SCG and ECG can assess CPX oxygen uptake and thereby classify clinical status for patients with HF.
Kaneko et al., 2022	OBS	PPG, ECG	Pulmonary congestion, HR, CAB	CABs related to S2 and S3 showed significant correlations with absolute pulmonary artery pressure values both at baseline and after exercise in patients with HF, but no significant correlations between their changes from baseline to post-exercise were observed in this study population
Di Rienzo et al., 2020	POC	PPG, ECG, SCG	HR, HRV, PTT	SCG and PTT can be derived from the data to obtain additional information on cardiovascular characteristics.
Savoldelli et al., 2022	POC	PPG, ECG, Thermometer	HR, Rhythm, Temperature	Medical personnel defined the methodology to efficiently monitor HF patients. Indeed, both parameters acquisition and televisits have been useful to make a complete patients' evaluation..

Abbreviations: OBS: Observational, RCT: Ransomized controlled trial, PT: Pilot trial, POC: Proof-of-Concept study, ECG: Eclectrocardiography, PPG: Photoplethysmography, SCG: Seismocardiography, PA: Physical activity, HR: Heart rate, SpO2: Oxygen saturation, PTT: Pulse transit time, RV, Heart rate variability, RR: Blood pressure, CAB: Cardiac acoustic biomarker, EE: Energy expenditure, (C)HF: (Chronic) Heart failure, NYHA class: New York Heart Association class, (HR)QOL: (Health-related) quality of life, 6-MWT: 6-Minute walking test, HFrEF: Heart failure with reduced ejection fraction, HFpEF: Heart failure with preserved ejection fraction, MDD: minimum detectable difference, MCID: minimum clinically important difference, KCCQ: Kansas City Cardiomyopathy Questionnaire, CPX: Cardiopulmonary exercise, ReDS: Remote dielectric sensing, RFU: , PCWP: Pulmonary capillary wedge pressure, WCD: Wearable cardioverter defibrillator, GDMT: Guideline directed medical therapy, ICD: Implantable cardioverter defibrillator, MLHFQ: Minnesota Living with Heart Failure Questionnaire, EMAT: electromechanical activation time, EF: Ejection fraction

Supplementary Table 2. Explanations of different measurement techniques

Type of device	***Explanation***
Accelerometers	Accelerometers, typically worn on the wrist, or hip, measure acceleration in one or multiple directions over time, translating this data into estimates of physical activity. While simple pedometers measure acceleration in a single direction, more precise devices measure linear acceleration in three directions (triaxial). The collected data can be used to generate axial acceleration counts or be reported as a step count. (1)
Bio-impedance	Bio-impedance measures the resistance of different tissues (e.g. fat, fluid and muscle) to through the flow of a small electrical current. Different tissues have various electrical resistance, therefore bio-impedance can be used to estimate the amount of fluid in the body. In the context of pulmonary fluid status, by using bio-impedance spectroscopy, impedance at different frequencies, detailed information about the fluid distribution can be obtained.(2,3)
ECG-based devices	Wearable devices incorporating ECG sensors are designed to measure various aspects of cardiac activity, including heart rate, heart rhythm, and heart rate variability (HRV). The established benchmark for these measurements is the electrocardiogram (ECG). A typical ECG entails the recording of the heart's electrical depolarization using three or more electrodes for a variable duration, typically spanning from 1 to 14 days. The data collected by ECG monitors is transmitted wirelessly or via device interrogation to a central server, where it undergoes analysis either through manual review, semi-automated processes, or machine learning techniques to produce a comprehensive report.(4)
PPG-based devices	Photoplethysmography (PPG)-based sensors measure blood volume changes by continuously emitting a pulse of photons through the skin. The variations in the intensity of reflected photons provide valuable information about the heart rhythm, offering cycle-to-cycle changes in cardiac hemodynamics. PPG relies on a light source, typically a light-emitting diode emitting green, red, or infrared light, to illuminate the skin's surface, making it a particularly suitable technique for wearables.(5)
SCG-based devices	A seismocardiogram (SCG) is the recording of chest wall vibrations in response to the movement of the heart and is associated with the movement of blood in the heart and the ejection of blood through the aorta. Additionally, SCG captures cardioacoustic markers such as electromechanical activation time (EMAT), left ventricular systolic time (LVST), S3 and S4 strengths, and systolic dysfunction index (SDI) which could be utilized for HF monitoring. (6)
Other techniques	Other techniques included in this review are thermometers for measuring skin temperature, humidity sensors for measuring skin humidity or potassium blood content, and microphones for measuring lung sounds and cardiac acoustic biomarkers.

(1) Liu F, Wanigatunga AA, Schrack JA. Assessment of Physical Activity in Adults Using Wrist Accelerometers. Epidemiol Rev. 2022 Jan 14

(2) Sanchez-Perez JA, Berkebile JA, Nevius BN et al. A Wearable Multimodal Sensing System for Tracking Changes in Pulmonary Fluid Status, Lung Sounds, and Respiratory Markers. Sensors (Basel). 2022 Feb 2

(3) Orea-Tejeda, A., Gómez-Martínez, M., González-Islas, D. et al. The impact of hydration status and fluid distribution on pulmonary function in COPD patients. Sci Rep 12, 1216 (2022).

(4) Krittanawong, C., Rogers, A.J., Johnson, K.W. et al. Integration of novel monitoring devices with machine learning technology for scalable cardiovascular management. Nat Rev Cardiol 18, 75–91 (2021)

(5) Reisner, A., Shaltis, P. A., McCombie, D. & Asada, H. H. Utility of the photoplethysmogram in circulatory monitoring. Anesthesiology 108, 950–958 (2008)

(6) Shandhi MMH, Fan J, Heller JA, et al. Estimation of Changes in Intracardiac Hemodynamics Using Wearable Seismocardiography and Machine Learning in Patients With Heart Failure: A Feasibility Study. IEEE Trans Biomed Eng. 2022 Aug;
